# Impact of Ga-68-PSMA PET/CT on management in prostate cancer patients with very early biochemical recurrence after radical prostatectomy

**DOI:** 10.1007/s00259-018-4249-z

**Published:** 2019-01-08

**Authors:** Usman Bashir, Alison Tree, Erik Mayer, Daniel Levine, Chris Parker, David Dearnaley, Wim J. G. Oyen

**Affiliations:** 1 Division of Radiotherapy and Imaging, Centre for Cancer Imaging/SRD, The Institute of Cancer Research, 15 Cotswold Road, London, Sutton SM2 5NG UK; 2 Department of Nuclear Medicine, The Royal Marsden NHS Foundation Trust, London, UK; 3 Department of Uro-Oncology, The Royal Marsden NHS Foundation Trust, London, UK; 4 The Royal Marsden NHS Foundation Trust, Department of Urology & Department of Surgery & Cancer, Imperial College London, London, UK

**Keywords:** PSMA-PET/CT, Prostate cancer, Recurrence

## Abstract

**Purpose:**

With the availability of ultra-sensitive PSA assays, early biochemical relapse (eBCR) of prostate cancer is increasingly being detected at values much lower than the conventional threshold of 0.2 ng/ml. Accurate localisation of disease in this setting may allow treatment modification and improved outcomes, especially in patients with pelvis-confined or extra-pelvic oligometastasis (defined as up to three pelvic nodal or distant sites). We aimed to measure the detection rate of [68]Ga-PSMA-HBNED-CC (PSMA)-PET/CT and its influence on patient management in eBCR of prostate cancer following radical prostatectomy (RP).

**Methods:**

We retrospectively identified 28 patients who underwent PSMA-PET/CT for post-RP eBCR (PSA < 0.5 ng/ml) at our tertiary care cancer centre. Two nuclear medicine physicians independently recorded the sites of PSMA-PET/CT positivity. Multidisciplinary meeting records were accessed to determine changes in management decisions following PSMA-PET/CT scans.

**Results:**

The mean age of patients was 65.6 years (range: 50–76.2 years); median PSA was 0.22 ng/ml (interquartile range: 0.15 ng/ml to 0.34 ng/ml). Thirteen patients (46.4%) had received radiotherapy in the past. PSMA-PET/CT was positive in 17 patients (60.7%). Only one patient had polymetastasis (> 3 sites); the remainder either had prostatectomy bed recurrence (*n* = 2), pelvic oligometastasis (*n* = 10), or extra-pelvic oligometastasis (*n* = 4). PSMA-PET/CT resulted in management change in 12 patients (42.8%), involving stereotactic body radiotherapy (*n* = 6), salvage radiotherapy (n = 4), and systemic treatment (*n* = 2).

**Conclusions:**

Our findings show that PSMA-PET/CT has a high detection rate in the eBCR setting following RP, with a large proportion of patients found to have fewer than three lesions. PSMA-PET/CT may be of value in patients with early PSA failure, and impact on the choice of potentially curative salvage treatments.

## Introduction

Despite advances in surgical technique, post-prostatectomy biochemical relapse (BCR) remains a significant problem, with 20% to 30% patients experiencing prostate-specific antigen (PSA) failure following radical prostatectomy (RP). A proportion of these patients harbour a low disease burden within the pelvis or extrapelvic oligometastases, especially during the early stages of BCR [1, 2]. Numerous studies have shown the advantage of early intervention in BCR when disease burden is low, and it has been shown that there is a loss of 2.6% biochemical control per 0.1 ng/ml rise in PSA [3]. For patients undergoing PSA monitoring, a PSA threshold of 0.2 ng/ml has been proposed to offer prostate bed salvage radiotherapy (SRT), with more recent evidence advocating SRT even earlier — at first sign of detectable PSA — with improved long-term disease control [3–5]. Following empirical SRT for BCR, a proportion of patients still relapse due to occult oligometastatic disease outside the prostate bed [6, 7]. Hence, more sensitive detection methods to localise individual disease sites could allow personalised treatment in early BCR (eBCR), defined here as PSA < 0.5 ng/ml.

Conventional imaging modalities used to detect prostate cancer include CT, bone scan, MRI, and more recently, choline-PET/CT. Although choline-PET/CT is widely considered the most sensitive of these tests, they are all typically negative at low PSA values, with most guidelines not recommending any of these modalities at PSA < 2 ng/ml [8]. Recently, Ga-68 labeled (Glu-NH-CO-NH-Lys-(Ahx)-[Ga-68(HBED-CC)] prostate-specific membrane antigen (PSMA) PET/CT has emerged as a more promising imaging modality in prostate cancer detection. Compared with choline-PET/CT, lymphadenectomy series have quantified its sensitivity and specificity as 65.9% and 98.9%, versus the reported sensitivity and specificity respectively of 49.2% and 95% for choline-PET/CT [8]. In one study, PSMA-PET/CT detected lesions in 44% patients with negative choline-PET/CT scans [8].

The detection rate of Ga-68-PSMA-11 PET/CT and its impact on patient management has been reported in a number of studies. The majority of the previous studies comprised heterogeneous patient populations including patients undergoing baseline staging mixed with patients with BCR in the post-RP and post-RT settings, over a wide range of PSA levels [9–11]. Only limited data are available on the impact of PSMA-PET/CT on the management of post-RP eBCR [7, 12]. One study reported detection of extra-pelvic oligometastases in 12.2% of patients in this setting [13]. Due to the potential benefit of targeted treatment of oligometastatic relapse detected by the previous generation of imaging studies [14], estimation of the efficicacy of PSMA-PET/CT in detecting oligometastases will support the design of therapeutic trials aimed at measuring long-term outcomes of these patients.

The primary objective of our study was to evaluate the proportion of patients with post-RP eBCR who were diagnosed with oligometastatic recurrence outside the prostatectomy bed following Ga-68-PSMA-11 PET/CT; the secondary objective was to document changes in patient management as a result of Ga-68 PSMA-11 PET/CT examinations.

## Materials and methods

Between 2015 and 2017, 152 patients underwent Ga-68 PSMA-11 PET/CT for BCR following radical prostatectomy, and had recorded multidisciplinary team (MDT) management plans. Patients were excluded if their PSA was > 0.5 ng/ml. As a result, 122 patients were excluded and the final cohort included 28 patients, none of whom had recently received abiraterone (i.e., within the past 12 months). If a patient had undergone more than one PSMA-PET/CT scan, his first scan was used for the study. The study proposal was approved by the Committee for Clinical Research of the Royal Marsden Hospital (SE705).

### Image acquisition

Ga-68-labelled PSMA-11 was obtained commercially from Mallinckrodt/Curium Pharma (London, UK). Patients were injected intravenously with a median dose of 126 MBq Ga-68 PSMA–11 (range 106–154 MBq) and, 60 minutes after injection, were imaged from the base of the skull to midthighs using a Gemini PET/CT scanner (Philips Medical Systems, Cleveland, OH, USA). Data were acquired for 3.0 min per bed position following low-dose CT scan (120 kV, 50mAs) for attenuation correction. PET data sets were reconstructed using ordered subset expectation maximization iterative reconstruction incorporating time-of-flight (three iterations and 33 subsets). Data were corrected for randoms, scatter, and attenuation; matrix size was 144 × 144 (4 mm pixel spacing).

### Lesion analysis

Ga-68-PSMA PET/CT scans were independently interpreted on a Hermes hybrid viewer workstation (Hermes Medical Solutions, Stockholm, Sweden) by two nuclear medicine physicians, in line with published guidelines [15]. Ga-68-PSMA uptake was quantified in terms of SUV_max_. Any focal uptake greater than background not attributable to physiologic activity was considered positive for malignancy, and correlated with low-dose CT for morphologic findings. In the literature, oligometastatic disease has been defined variably in terms of number and sites of lesions [1, 13, 16]. We defined oligometastasis as ≤ 3 N1 or M1a lesions [17]. Lesion numbers and sites were recorded per patient as prostatectomy-bed recurrence, pelvic oligometastasis, extra-pelvic oligometastasis (without or in addition to pelvic lesions), and polymetastasis (> 3 lesions).

### MDT decision review

We retrospectively accessed patient records to review management decisions undertaken based on MDT discussion of each case. At our institution, patients do not routinely undergo pelvic lymph node dissection at the time of radical prostatectomy. The standard of care of eBCR following radical prostatectomy, in the absence evidence of disease outside the prostatectomy bed, comprises prostatectomy bed radiotherapy.

The impact of PSMA-PET/CT on management was measured as the proportion of patients whose treatment was changed from a previous plan. Management options were categorised as PSA monitoring, androgen deprivation treatment (ADT; with or without chemotherapy), localised treatment of oligometastases (surgery or sterotactic body radiotherapy [SBRT]), and SRT.

### Statistical analysis

For continuous variables, means and standard deviations are reported. PSMA-PET positive and negative groups were compared in terms of PSA levels using the Wilcoxon test, and in terms of management decision using Fisher’s exact test. The level of significance was set at 0.05. All statistical analysis was done using R version 3.3.5 [18].

## Results

We identified 28 patients with mean age 65.6 years (range 50–76.2 years) and PSA median 0.22 ng/ml (interquartile range: 0.15–0.34). Six patients had received ADT in the past (i.e., more than 12 months before PSMA-PET imaging). Thirteen patients had also received radiotherapy (prostatectomy bed only, *n* = 11; prostatectomy bed plus pelvic lymph nodes, *n* = 2) adjuvantly (n = 1) or as salvage treatment for a previous BCR (*n* = 12). Clinical characteristics of the 28 patients are presented in Table 1.

**Table 1:** Clinical and pathologic characteristics of the study population (*n* = 28)

Clinical variable	Value
Mean age (years)	65.6 (range 50–76.2)
PSA at time of assessment (ng/ml)^a^	
Mean	0.24 (SD 0.12)
Median	0.22 (IQR 0.15–0.34)
PSA pre-prostatectomy (ng/ml)	
Mean	11 (SD 9.07)
Median	7.6 (IQR 5.7–11.35)
Gleason score	
6–7	21 (75%)
8–10	7 (25%)
Tumour stage	
T2	7 (25%)
T3	21 (75%)
Nodal stage	
N0	18 (64%)
N1	4 (14%)
Nx	6 (22%)
Positive margin	
R0	14 (50%)
R1	8 (29%)
Unknown	6 (21%)
NCCN risk group	
Intermediate	3 (11%)
High	23 (82%)
Unknown	2 (7%)
Previous androgen dDeprivation treatment ^b^	6 (21.4%)
Previous radiotherapy	
Prostatectomy bed only	11 (39.2%)
Prostatectomy bed and pelvic nodes	2 (7.1%)

^a^PSA-value at the time of referral for PSMA-PET/CT

**Fig. 1:**
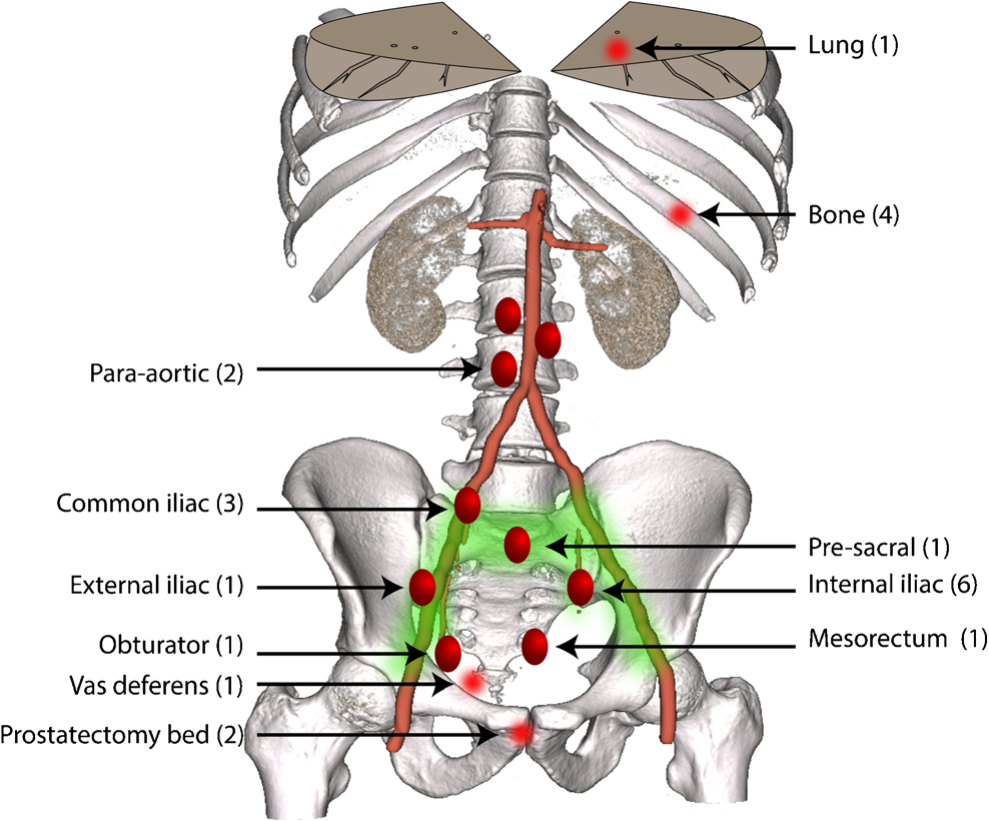
Site-wise distribution of individual lesions in 16 patients with prostatectomy bed relapse or oligometastasis; the one patient with polymetastasis is not included in this analysis. *Green-shaded region* shows typical pelvic lymph node radiotherapy fields. The lymph node short axes were median 5 mm (range 3 mm to 8 mm)

**Fig. 2:**
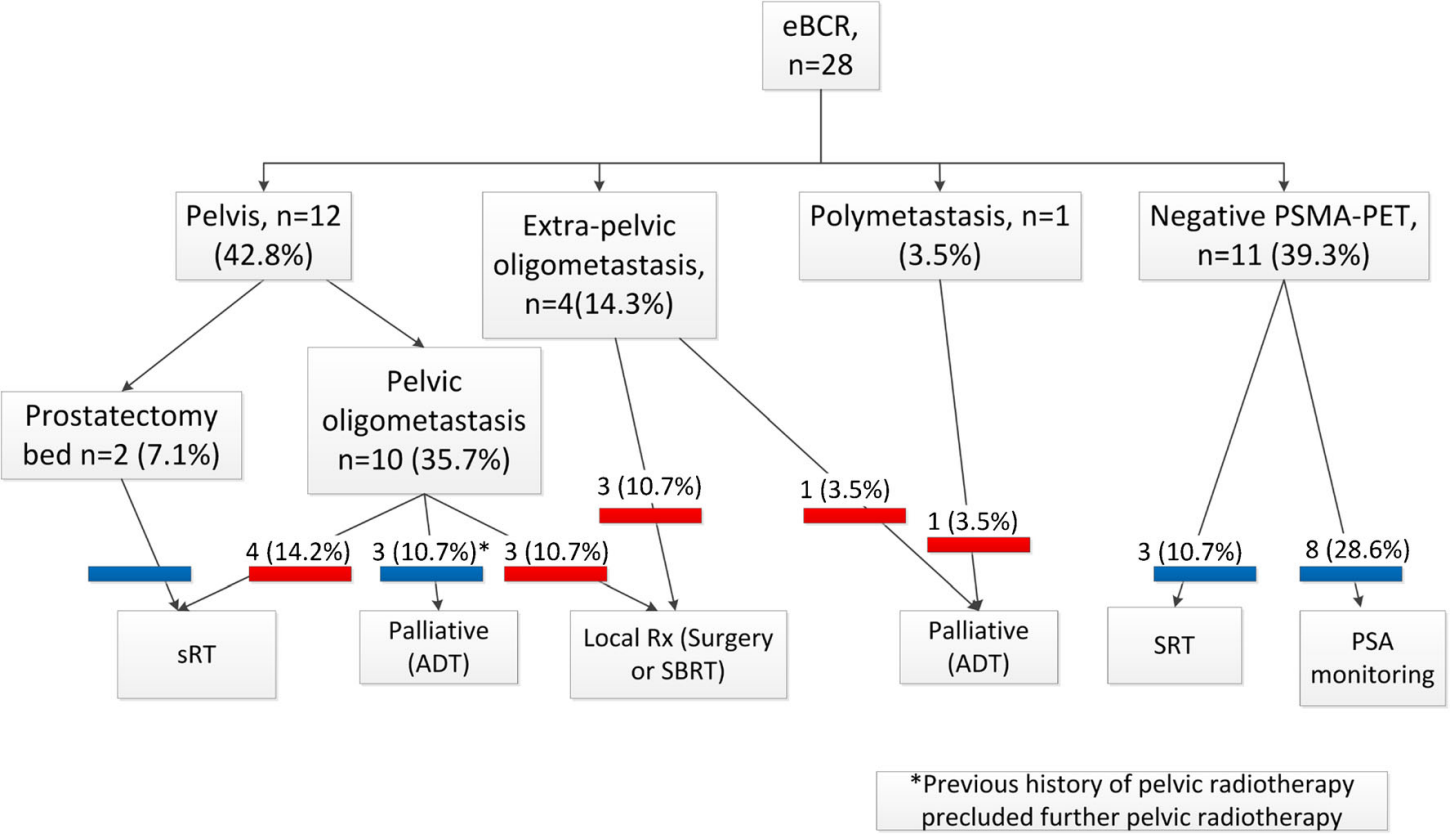
Flowchart illustrating sites of disease and management plans. *Bars above the management plans* show whether PSMA-PET/CT changed management (*red*) or not (*blue*). *eBCR* = early biochemical recurrence. *SRT* = salvage radiotherapy. *ADT* = androgen deprivation treatment. *Rx* = therapy

PSMA-PET/CT was positive in 17 patients (60.7%) and negative in 11 (39.3%). PSA values in the positive and negative categories were similar (mean 0.26 ± 0.14 and 0.23 ± 0.15, respectively; *p* = 0.57). Comparing PSMA-negative and PSMA-positive groups, adopted treatment strategies were significantly different (Fisher exact test; *p* < 0.0001). Figure 1 provides a graphical overview of sites of PSMA-PET positivity. Figure 2 illustrates site-wise breakdown of treatment plans. In summary, all patients with PSMA-PET/CT positive findings underwent treatment, whereas only three of 11 patients with negative scans were actively treated, the remainder (*n* = 8) undergoing continued PSA monitoring with a plan to repeat PSMA-PET after a short-term follow-up.

PSMA-PET/CT resulted in a change of management in 12 of 28 (42.8%) patients (red coloured bars in Fig. 2): There was no change in management of patients with a negative PSMA-PET/CT. Of the ten patients with pelvic oligometastases, four patients received extended pelvic SRT (including pelvic lymph nodes). The original plan in three of these patients had been to irradiate the prostatectomy bed only, and in one patient (PSA 0.1 ng/ml), to continue PSA monitoring. In three patients with pelvic oligometastases, ADT was instituted and PSMA-PET/CT did not change the treatment plan; all three patients had received pelvic SRT due to a previous relapse, and further pelvic irradiation could not be offered due to the risk of toxicity. All four patients with extra-pelvic oligometastases had a change in management plan based on the detection of extra-pelvic disease. Whereas three patients received site-specific treatment (surgery or SBRT), one patient with extra-pelvic bone metastasis was not offered ablative treatment and management was changed to ADT.

Past radiotherapy also seemed to have an impact on sites of PSMA-PET positivity: Neither of the patients with prostatectomy bed recurrence had a history of prostate bed radiotherapy. Of the ten patients with pelvic oligometastasis, six patients had received prostate bed only radiotherapy, and one patient with a sacral bone metastasis had previously received prostatectomy bed and pelvic lymph node radiotherapy. Hence, none of the patients with prior history of radiotherapy had in-field recurrence.

### Follow-up of patients after PSMA-PET-directed management

All patients with positive PSMA-PET/CT (*n* = 17) were given either long-term palliative ADT or short-term ADT alongside oligometastasis treatment. In all treated patients, PSA fell to undetectable levels. However, two patients — both recipients of PSMA-guided SBRT — experienced a further relapse within 6 months: one patient had a PSMA-avid lesion in the vas deferens which was surgically excised, but subsequently relapsed at the same site. Regarding the other patient, a PSMA-positive common iliac lymph node was treated with SBRT and ADT. After an initial drop, his PSA rose from 0.4 ng/ml at initial PSMA-PET/CT to 1.17 ng/ml over the next 6 months, possibly due to occult disease sites at the time of the initial PSMA-PET/CT scan. However, subsequent imaging did not detect the site(s) of relapse over 13 months of follow-up from the end of SBRT.

**Fig. 3:**
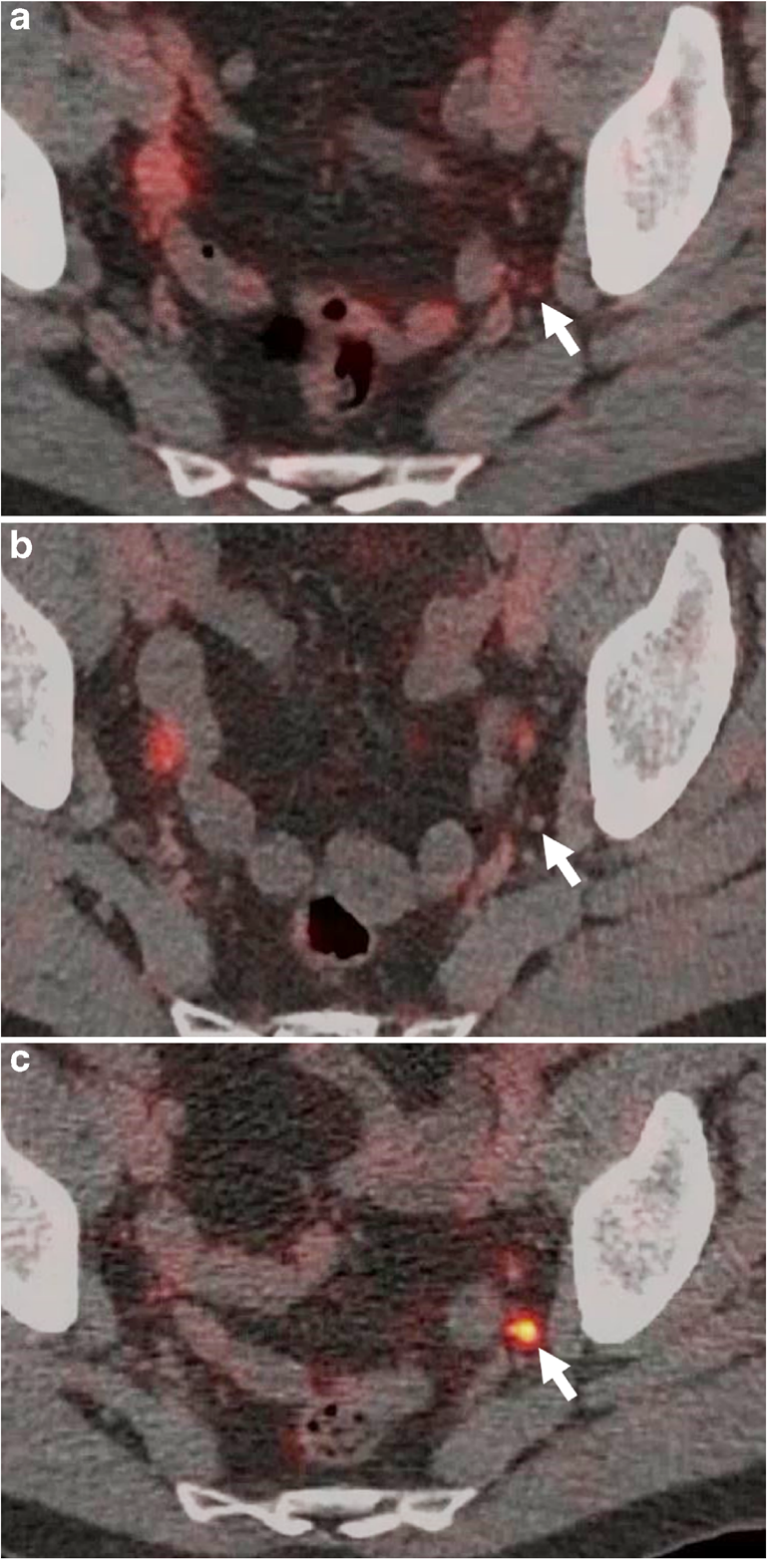
Serial Ga-68-PSMA-PET/CT in a 67-year-old man with BCR following radical prostatectomy and prostatectomy bed radiotherapy. **a** Initial scan at PSA 0.1 ng/ml. Left internal iliac lymph node (*arrow*) considered benign, morphologically (< 4 mm diameter) and metabolically (SUV 1.2). **b** PSA rise to 0.2 ng/ml, follow-up scan after 4 months. Minimal enlargement of the lymph node, still within normal limits (SUV 0.7; PSA). **c** Further PSA rise to 0.4 ng/ml, follow up scan 5 months after *b*. Lymph node remains subcentimetre (6 mm), but due to high PSMA uptake (SUVmax 8.2), now considered suspicious for malignant metastasis

In all patients with a negative PSMA-PET/CT scan who received treatment (SRT; *n* = 3), PSA became undetectable. Of the patients with negative PSMA-PET/CT on PSA monitoring (*n* = 8), PSA became undetectable in one patient (PSA at time of imaging 0.07 ng/ml) and continued to rise in seven patients. In one of these patients with negative PSMA-PET/CT (PSA at time of initial imaging 0.1 ng/ml), a third serial Ga-68 PSMA–11 scan became positive when PSA rose to 0.4 ng/ml, showing a pathological pelvic lymph node (Fig. 3). This patient was then given SRT. The remaining six PSMA-PET/CT negative patients are still undergoing PSA monitoring with a median follow-up period of 11 months (range 5–17 months) at the time of writing.

## Discussion

With few exceptions, the majority of studies reporting the detection rates of PSMA-PET/CT in BCR involve heterogeneous patient cohorts including patients who have recently received ADT before PSMA-PET, have undergone either RP or RT, or have a wide range of PSA with relatively few cases in the eBCR category [9–11]. Conventional imaging may also detect disease when the PSA levels are more elevated — being associated with higher disease burden. The incremental benefit of PSMA-PET/CT over conventional imaging remains to be established at higher PSA values. Reviewing PSMA-PET/CT detection rates in the eBCR sub-category of previous reports, our detection rate of 57% falls within the published range of 45% to 60% [7, 9, 10, 12, 19–22].

Our trend of disease-localisation is similar to that reported in recent studies in the early BCR (PSA < 1 ng/ml) setting in post-RP patients [12, 13]. For example, Calais et al. reported pelvis-confined disease in 99 (36.7%), extra-pelvic oligometastases in 33 (12.2%), and polymetastases in six patients (2.2%) [13]. Their definition of oligometastases included extra-pelvic sites only. In another study by Emmett et al. [12], the authors reported pelvis-confined disease in 79 of 164 patients (48%) and distant metastases in 23 (14%), the remainder (*n* = 62; 38%) being negative. The authors of this study did not further characterise disease burden in terms of polymetastases and oligometastases; therefore, the frequency of oligometastasis in their cohort cannot be commented on. An important departure between our study and both these studies is the much higher frequency of prostatectomy bed recurrence of 17.5% and 23% reported by Calais et al. and Emmet et al. respectively, compared with only 7.1% (*n* = 2) in our study. We believe this difference is because 11 of our patients had received prostatectomy-bed radiotherapy in the past, and a further two had undergone prostatectomy-bed and lymph node radiotherapy, which would have arguably lowered the risk of future relapses at these sites. We suspect that the actual proportion of prostatectomy-bed relapse is even higher than that reported by these two groups, since prostatectomy-bed activity is likely to be masked due to the high urine concentration of PSMA in the bladder [23].

In a recent study on a homogeneous cohort (*n* = 119) of patients with early post-RP BCR (PSA 0.2–0.5 ng/ml), Farolfi et al. reported a detection rate of 34% [24]. Unlike some patients in our study, none in their cohort had received SRT in the past. Nevertheless, the detection rate was much lower, despite the absence of previous SRT which might have sterilised the pelvic sites and lowered the probability of pelvic recurrence. It is possible that since a proportion of their patients had received ADT (7.6% at the time of imaging and 23.5% during recurrence), PSMA expression was suppressed in a number of cases, leading to a lower detection rate.

 Regarding the negative results in 11 patients (39.3%), we believe that PSMA-PET/CT should be considered false negative in most cases, although a proportion of patients may have low PSMA expressing disease. It has been previously pointed out that despite its superior sensitivity compared to other imaging modalities, detecting a very low disease burden remains challenging for PSMA-PET/CT, especially when lesions are < 4 mm, as exemplified in Fig. 3 [15]. Although PSMA is over-expressed in all prostate cancer cases, the intensity of over-expression varies, and around 50% of tumours show relatively low degrees of PSMA over-expression and correspondingly lower SUVs on PSMA-PET/CT, irrespective of PSA levels [25–27]. The significance of a negative PSMA-PET/CT has been highlighted recently by Emmett et al., who showed high PSA-response rates (85%) to SRT in patients with negative PSMA-PET/CT, suggesting pelvis-confined disease in the majority of patients with a negative PSMA-PET/CT, especially within the prostatectomy bed, which may be obscured due to the very close proximity to high urinary PSMA activity [12].

Our retrospective study has several limitations: 13 out of 28 patients in our relatively small cohort had received SRT previously. This could have potentially skewed the detection rates in favour of extra-pelvic sites. Secondly, we did not have definitive pathologic proof of disease positivity in the majority of our patients, who were all imaged in the course of routine clinical assessment. Referrers proceeded with treatment based on clinical judgement and reported specificity of PSMA; thus, pathological confirmation was not obtained in most cases [8]. Obviously, PSA-response cannot be used to confirm the efficacy of PSMA-PET/CT guided local treatment, since most of our patients received ADT. Follow-up of our cohort continues and long-term outcomes of patients with PSMA-PET/CT positive versus negative will be assessed at a later date.

## Conclusion

Our study shows that PSMA-PET/CT has a clinically significant rate of prostate cancer detection in post-RP patients with eBCR despite very low PSA levels. PSMA PET/CT suggested limited oligometastatic disease in half of the patients in our cohort. Given the increasingly lower threshold for active treatment of BCR, PSMA-PET/CT is uniquely advantageous in influencing management of these patients. However, whether this change in management, i.e., PSMA-guided alteration in SRT fields or ablative treatment of oligometatic sites, translates into improved long-term outcome needs to be investigated in prospective studies.
